# High-Sensitivity and In Situ Multi-Component Detection of Gases Based on Multiple-Reflection-Cavity-Enhanced Raman Spectroscopy

**DOI:** 10.3390/s24175825

**Published:** 2024-09-07

**Authors:** Dewang Yang, Wenhua Li, Haoyue Tian, Zhigao Chen, Yuhang Ji, Hui Dong, Yongmei Wang

**Affiliations:** 1 College of Ocean Science and Engineering, Shandong University of Science and Technology, Qingdao 266590, China; yangdewang_lcu@126.com (D.Y.); wh1463425962@163.com (W.L.); 2 Faculty of Information Science and Engineering, Ocean University of China, Qingdao 266100, China; thaoyue1999@163.com; 3 College of Safety and Environmental Engineering, Shandong University of Science and Technology, Qingdao 266590, China; 15166344354@163.com (Z.C.); ji3282466882@163.com (Y.J.); 4 Institute of Machinery Manufacturing Technology, China Academy of Engineering Physics (CAEP), Mianyang 621900, China; dh_caep@163.com

**Keywords:** high sensitivity, multi-component detection, multiple-reflection cavity, Raman spectroscopy

## Abstract

Raman spectroscopy with the advantages of the in situ and simultaneous detection of multi-components has been widely used in the identification and quantitative detection of gas. As a type of scattering spectroscopy, the detection sensitivity of Raman spectroscopy is relatively lower, mainly due to the low signal collection efficiency. This paper presents the design and assembly of a multi-channel cavity-enhanced Raman spectroscopy system, optimizing the structure of the sample pool to reduce the loss of the laser and increase the excitation intensity of the Raman signals. Moreover, three channels are used to collect Raman signals to increase the signal collection efficiency for improving the detection sensitivity. The results showed that the limits of detection for the CH_4_, H_2_, CO_2_, O_2_, and N_2_ gases were calculated to be 3.1, 34.9, 17.9, 27, and 35.2 ppm, respectively. The established calibration curves showed that the correlation coefficients were all greater than 0.999, indicating an excellent linear correlation and high level of reliability. Meanwhile, under long-time integration detection, the Raman signals of CH_4_, H_2_, and CO_2_ could be clearly distinguished at the concentrations of 10, 10, and 50 ppm, respectively. The results indicated that the designed Raman system possesses broad application prospects in complex field environments.

## 1. Introduction

With the development of the global economy and the acceleration of industrialization, people’s lives have been greatly facilitated and improved [1]. However, this has also brought about the need for gas emission detection and gas leak accident investigation in various fields, such as the electrical industry, coking plants, and the petrochemical industry [2,3]. Therefore, the development of gas detection technology to quickly and accurately analyze various gases in different industrial scenarios has become an urgent demand to realize energy exploration, environmental monitoring [4,5], etc.

The traditional gas measurement methods mainly include chemical reaction methods [6], electrochemical methods [7], gas chromatography [8], mass spectrometry [9], etc. The significant advantage of the chemical reaction methods and electrochemical methods is the high detection sensitivity for the targeted gases; however, they are susceptible to environmental interference [7]. Gas chromatography and mass spectrometry possess the advantages of high sensitivity and precise resolution, and they have been widely used in laboratory tests [9]. Meanwhile, the common carrier gases used in gas chromatography are hydrogen (H_2_), nitrogen (N_2_), and helium (He), which will cause interference for the detection of H_2_ and N_2_ [10]. Moreover, the manual sampling and pre-treatment of the gas samples are necessary for the traditional gas detection methods, which are major limitations for the quantitative analysis of industrial gases in complex field environments [11,12,13]. Compared with the traditional methods, the spectroscopic methods do not require the pre-treatment process, showing the advantages of fast, non-invasive, efficient, and dynamic detection ability [12]. Furthermore, they are suitable for in situ rapid detection and continuous real-time online analysis, becoming a hot topic of research and application in many different areas [12,13,14,15]. The most common spectroscopic methods for gas detection include infrared spectroscopy [14,16] and Raman spectroscopy [17,18]. Based on the analysis principle, Raman spectroscopy could analyze the molecular vibration and rotational energy changes in the targeted gases [19,20]. Different gas samples have their unique molecular structures, enabling the establishment of Raman spectra for the identification and quantitative analysis of the components [21,22]. Therefore, as a non-destructive spectral technique, Raman spectroscopy technology occupies a very important position in in situ gas detection technology.

Considering the low frequency of Raman scattering, the Raman signal strength is relatively weak, making it difficult to be captured and analyzed in actual gas detection [22,23]. To overcome these disadvantages, many studies have been involved in the approaches to improve the detection sensitivity, such as surface-enhanced spectroscopy, tip-enhanced spectroscopy, resonance spectroscopy, and cavity-enhanced spectroscopy [24]. However, surface-enhanced spectroscopy and tip-enhanced spectroscopy require strict experimental equipment and test conditions, making them unsuitable for real-time and on-site exploration [25]. Resonance spectroscopy could enhance the Raman signals of one substance at a time; however, it is difficult to enhance the Raman signals of multiple substances at the same time. In order to achieve the monitoring of multi-component gases, multiple-reflection-cavity-enhanced Raman spectroscopy technology has been developed to enhance the Raman signals [13,16]. The principle is to increase the amount of reflection in the cavity to enhance the absorption and scattering intensity of the lights. In addition, the multiple-reflection cavity could focus the lights at one point to increase the lights’ intensity and excitation efficiency. Li et al. [26] proposed a near-concentric-cavity Raman enhancement method where two opposing concave mirrors form a confocal cavity. Benefiting from the large reflection times (50 times), the LOD values of CO_2_, C_2_H_4_, N_2_, C_2_H_2_, and H_2_ could be decreased to 36, 18, 49, 12, and 28 ppm, respectively [26]. Utsav et al. [27] described a multiple-reflection cavity with 120 reflections to increase the Raman scattering intensity, exhibiting an increase in the scattering intensity by 83 times compared with that of the single pass of lights. Furthermore, Yang et al. [28] used two concave mirrors to form a near-concentric multiple-reflection cavity, which could largely increase the Raman signal intensity with lower LOD values of CO_2_ (51.97 μL·L^−1^), CH_4_ (9.9 μL·L^−1^), C_2_H_6_ (14.1 μL·L^−1^), and C_2_H_8_ (15.9 μL·L^−1^), respectively. Muller et al. [29] designed a near-concentric multiple-reflection cavity to collect most of the Raman scattering signals to achieve an LOD of 0.1 μL·L^−1^ for H_2_.

Herein, based on the multiple-reflection-cavity-enhanced Raman spectroscopy technology, this paper presents the design and assembly of a multi-channel cavity-enhanced gas Raman spectroscopy system for in situ gas analysis. Based on our previous studies [13,15,16], we have further optimized the optical path structure. Firstly, we eliminated the original in-cavity sample cell and integrated the multiple-reflection cavity into a unified sample cell unit, effectively reducing the loss caused by the window plates. Subsequently, we reduced the focal length of the cavity mirrors in the multiple-reflection cavity to decrease the volume of the sample cell and increase the amount of reflections. Furthermore, we integrated the multiple-reflection cavity with a laser to enhance the stability of the system. Through these improvements in the optical path and mechanical structure, we have successfully increased the detection sensitivity of the system.

## 2. Experiment Setup

### 2.1. Cavity-Enhanced Raman Spectroscopy Principle

Raman scattering spectroscopy is an inelastic scattering process, and the scattering intensity could be calculated by Equation (1) [30]:

(1) $$I_{i}=k\Omega \frac{\partial \sigma }{\partial \Omega }n_{i}lI_{0}$$

where $I_{i}$ is the Raman scattering intensity, k is the scattering coefficient, Ω is the solid angle of signal collection, l is the effective length of lights in the sample, $\frac{\partial \sigma }{\partial \Omega }$ is the microscopic Raman scattering cross-section, $n_{i}$ is the concentration of the samples being tested, and $I_{0}$ is the power of the excitation light, respectively.

According to Equation (1), the intensity of Raman scattering is directly proportional to the intensity of the excitation light ($I_{0}$), the effective length (l), and the solid angle of signal collection (Ω). Thus, we could enhance the monitoring sensitivity by increasing these three parameters. In our previous studies, we utilized multiple-reflection cavities and resonant cavity enhancement techniques [13,15,16] to increase the intensity of the excitation light to improve the signal sensitivity. And, the hollow-core fiber technology was used to further enhance the signal sensitivity by increasing the effective interaction length [31]. However, these methods in the previous studies showed that the collection efficiency of Raman signals is not relatively high. To address this issue, this paper proposed an innovative approach by increasing the collection solid angle to further improve the signal collection efficiency. This strategy could effectively improve the efficiency of signal collection and raise the system’s detection sensitivity.

### 2.2. Parameter Design of Raman Spectroscopy System

**Figure 1:**
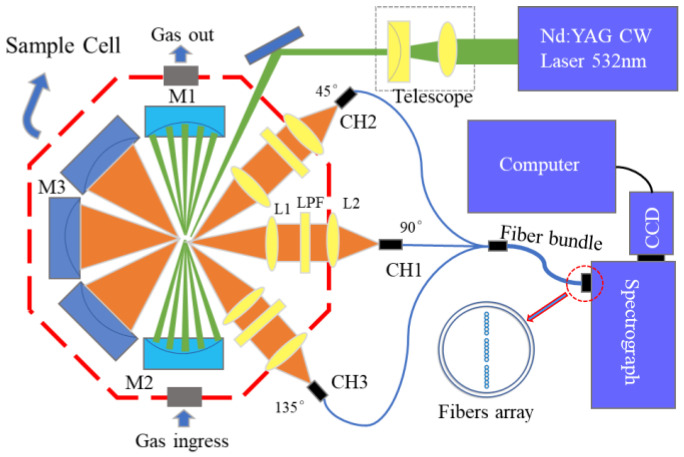
Schematic diagram of the Raman spectroscopy system and the optical path.

The overall schematic diagram of the Raman spectroscopy system is shown in Figure 1. The designed Raman spectroscopy system is ingenious and efficient, with its core component being the near-concentric-cavity reflector located in the sample pool. The system operates by emitting a laser from the laser source, which passes through a telescope system composed of plano-convex lens (focal length of 100 mm) and plano-concave lens (focal length of −50 mm), cleverly reducing the diameter of the laser beam by half. Subsequently, this laser beam was precisely directed by a total reflection mirror to the sample pool, where the near-concentric-cavity reflector significantly enhances Raman signals. The enhanced Raman scattered signal was efficiently coupled into optical fibers through three identical collection systems to be eventually converged into the spectrometer. The computer system would be responsible for capturing and processing these spectral signals to ensure the accurate analysis of the data.

As shown in Figure 1, the Raman spectroscopy system incorporates a 532 nm continuous-wave laser with a line width of 0.1 nm and a power output of 1 W. The laser beam emitted by the laser source was directed through a telescope system to narrow the beam to focus the laser beam at the heart of the multi-reflection cavity. This cavity is elegantly composed of two concave mirrors (M1 and M2) with a focal length of 20 mm and a diameter of 25 mm. The laser forms a near-concentric-cavity reflection pattern in the multi-reflection cavity with approximately 40 reflections. The Raman signals generated at the cavity’s center were collected into the optical fiber by a pair of achromatic lenses (L1 and L2). These achromatic lenses are of a focal length of 35 mm and a diameter of 25 mm. A 532 nm high-pass filter (LPF) was inserted between lenses L1 and L2 to effectively filter out the Rayleigh scatter signals. On the opposite side of the collection path, a concave mirror (M3) with a focal length of 20 mm and a diameter of 25 mm was strategically positioned 40 mm away from the cavity center, reflecting the Raman signals in this direction to enhance the signal collection efficiency. Achromatic lenses L1 and L2, high-pass filter LPF, and concave mirror M3 formed signal collection channel CH1. Similar to the components of CH1, two additional channels, including CH2 and CH3, were positioned at the angle of ±45° relative to the channel of CH1. The sample cell was encased in an octagonal shell with two ventilation holes, including a laser entry window and three signal collection windows. The signal collection fibers consisted of an 18-core fiber bundle (each core with a diameter of 200 μm), arranged linearly at one end of the spectrometer as depicted in Figure 1. On the other end, it was evenly divided into three bundles, each of which included the optical fiber with six cores. These optical fibers were also arranged linearly to collect the Raman signals from channels of CH1, CH2, and CH3, respectively. The spectrometer used in the system was a self-developed 532 nm Raman spectrometer with an entrance slit width of 50 μm, a groove density of 1800 line/mm, a focal length of 90 mm, and a resolution of 7 cm^−1^. The detector employed in the system was the iVac 316 from Andor Corporation (UK) with a pixel count of 2000 × 256 and a detection range of 1000 to 4500 cm^−1^.

### 2.3. Sample Preparation and Spectra Acquisition

In order to evaluate the performance of the Raman spectroscopy system, several gas mixtures with different components were configured in the experiment. Their components and concentrations are summarized in Table 1. For the 18 different gas samples, the composition of the gas mixture included hydrogen (H_2_), methane (CH_4_), carbon dioxide (CO_2_), oxygen (O_2_), nitrogen (N_2_), and argon gases (Ar), respectively. To evaluate the detection capabilities of the Raman system, we conducted experiments using this Raman system to detect the gas mixture. The experiment parameters were set as the laser power of 1 W, the integration time of 5 s, the cumulative number of 4, and the total acquisition time of 20 s per sample. By analyzing the spectral data of these gas samples, the detection capability of the designed Raman spectroscopy system was evaluated.

## 3. Results

### 3.1. Original Raman Spectra of Different Gases

**Figure 2:**
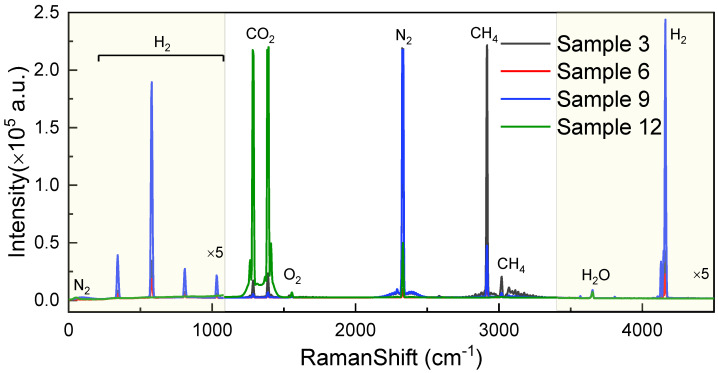
Original Raman spectra of the mixed gases (Nos. 3, 6, 9, and 12) with 5× magnification for the ranges of 0–1100 cm^−1^ and 3400–4400 cm^−1^.

Considering the Raman signals of the common gases, the detection range of the designed Raman spectroscopy system was distributed in the range of 500–4500 cm^−1^. The original Raman spectra of the gas samples of Nos. 3, 6, 9, and 12 are shown in Figure 2, where the Raman signals of H_2_ were the most widely distributed. The Raman peaks of H_2_ were located at 341 cm^−1^, 579 cm^−1^, 810 cm^−1^, 1030 cm^−1^, and 4156 cm^−1^, respectively [32]. In addition, there were other typical gas Raman signals, such as CO_2_ (1289 cm^−1^ and 1387 cm^−1^), O_2_ (1555 cm^−1^), N_2_ (2331 cm^−1^), and CH_4_ (2920 cm^−1^ and 3019 cm^−1^) [33]. Apart from the vibrational peaks, the rotational peaks of N_2_ and CH_4_ can also be clearly distinguished in Figure 2.

Moreover, Figure 2 shows the magnified view of the original Raman spectra in the ranges of 0–1100 cm^−1^ and 3400–4400 cm^−1^. The background intensity was approximately 2300, exhibiting a high signal-to-noise ratio. Compared to the Raman signals collected by backscattering, the Raman signals collected laterally had relatively lower stray light, which could be beneficial for the detection of trace gases. In order to explore the detection capability of the trace gases, we positioned the collection optical path at an angle to the excitation beam, as depicted in Figure 1. The angles for the three channels were set as 45°, 90°, and 135°, respectively. As shown in Figure 2, a relatively lower background intensity was obtained, benefiting the detection of the Raman signals of gases with low concentrations.

### 3.2. Raman Spectra of Gas Samples under Different Concentrations

**Figure 3:**
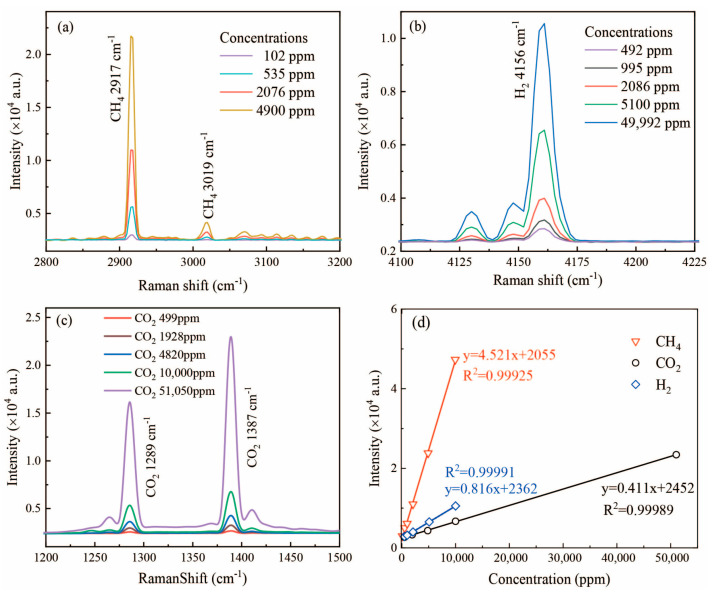
Raman spectra of different gas samples CH_4_ (**a**), H_2_ (**b**) and CO_2_ (**c**) under different concentrations and the calibration curves of gas concentration versus Raman peak intensity (**d**).

To explore the detection sensitivity of this Raman spectroscopy system for different gases, the gas samples (CH_4_, H_2_, and CO_2_) under different concentrations were detected to obtain the Raman spectra, as shown in Figure 3a–c. The Raman characteristic peaks of CH_4_, H_2_, and CO_2_ were located at 2917 cm^−1^/3019 cm^−1^, 4156 cm^−1^, and 1289 cm^−1^/1387 cm^−1^, respectively. The prepared concentrations of the gas samples were CH_4_ (102, 535, 2076, and 4900 ppm), H_2_ (492, 995, 2086, 5100, and 49,992 ppm), and CO_2_ (499, 1928, 4820, 10,000, and 51,050 ppm), respectively. Based on the Raman spectra of the gas samples, the Raman signals of CH_4_, H_2_, and CO_2_ at different concentrations were extracted, as shown in Figure 3a–c. It was clearly found that the intensity of the Raman signals increased with the increase in the gas concentration. The characteristic peak intensities of the gas samples at different concentrations were extracted and established the calibration curves (concentration versus peak intensity), as shown in Figure 3d. The points and the lines represent the peak intensities and linear regression of the gas characteristic peaks at different concentrations. The correlation coefficients of these three fitting lines were all greater than 0.999, indicating that the analysis method showed an excellent linear correlation and a high level of reliability [34]. 

### 3.3. Limit of Detection of the Raman System

To determine the limit of detection (LOD) of the system, the calculations of LOD were performed according to the definition proposed by the Applied Chemistry Association [35,36], as shown in Equation (2).

C_L_ = k·S_b_/m (2)

where C_L_ is the LOD, k is the confidence factor (3 was chosen in this article), S_b_ is the standard deviation of the blank sample, and m is the slope of the calibration curve in the low concentration range. The LODs of various gas components were calculated and summarized in Table 2.

As shown in Table 2, when the detection time was 20 s, the LODs of this Raman system were 3.14, 34.79, and 17.9 ppm for CH_4_, CO_2_, and H_2_, respectively. The LODs of the different gas samples varied in relation to the scattering cross-section of the gases and the detection efficiency of the instrument at the corresponding wavelength [36]. Compared with our previous research [13,15,16], the detection time of the Raman system in this study was largely reduced from 100 s to 20 s. Meanwhile, the LOD of CO_2_ decreased from 70 ppm to 34 ppm, showing a significant improvement in the detection ability. In addition, we applied the same signal analysis method to calculate the LODs of N_2_ and O_2_. The LODs of N_2_ and O_2_ were 26.96 and 35.17 ppm, indicating the advantages of high detection sensitivity and the simultaneous detection of multiple gas components of the Raman spectroscopy system.

### 3.4. Long-Time Integration Detection of the Raman System

**Figure 4:**
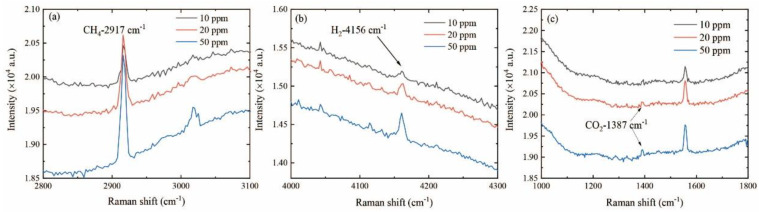
Raman spectra of low concentrations of CH_4_ (**a**), H_2_ (**b**), and CO_2_ (**c**) under long-time integration.

In order to further explore the LOD values of the system for different gases, we prepared a mixture of CH_4_, H_2_, CO_2_, and Ar, in which the origin concentrations of CH_4_, H_2_, and CO_2_ were 50, 50, and 50 ppm, respectively. Using a mass flow controller, this gas was mixed with Ar gas in different mass ratios to obtain the mixed gases with the concentrations of 10 ppm and 20 ppm. Furthermore, the detection parameters were optimized; thus, the detection time was extended to 100 s. The Raman spectra of the three gases at different concentrations are shown in Figure 4. As shown in Figure 4a, the Raman signals of CH_4_ at the concentration of 10 ppm could be clearly distinguished, exhibiting a relatively strong detection ability for CH_4_. Similar to the signal strength of CH_4_, the Raman signals of H_2_ at the concentration of 10 ppm could also be clearly distinguished, as depicted in Figure 4b. Compared with the detection of CH_4_ and H_2_, the Raman peak of CO_2_ at 10 ppm was difficult to be clearly distinguished, as shown in Figure 4c. The Raman signal strength of CO_2_ increased with the increase in the concentration of CO_2_. When the concentration of CO_2_ increased to 50 ppm, the Raman peaks of CO_2_ could be clearly distinguished. Therefore, increasing the integration time could largely benefit the detection ability. The LODs of the Raman systems for CH_4_ and H_2_ could reach below 10 ppm with an integration time of 100 s.

## 4. Conclusions

For the in situ multi-component detection of mixed gases, this paper developed a gas Raman system based on multiple-reflection-cavity-enhanced Raman spectroscopy technology. Based on the precision of the optical engineering and design, this system can ensure the highest efficiency and accuracy for Raman signal detection. Based on the qualitative and quantitative analyses of the mixed gases, a lower LOD could be achieved, greatly improving the accuracy of the gas detection. Meanwhile, the established calibration curves (concentration versus peak intensity) showed that the correlation coefficients were all greater than 0.999, exhibiting an excellent linear correlation and high level of reliability. After the long-time integration detection of the system, the Raman signals of CH_4_, H_2_, and CO_2_ could be clearly distinguished at the concentrations of 10, 10, and 50 ppm, respectively. This designed multiple-reflection-cavity-enhanced Raman spectroscopy system showed the advantages of higher sensitivity, lower environmental disturbance, and in situ multi-component detection. With the development of instruments and equipment technology, the portability of the designed gas Raman spectroscopy detection system in this study could be further enhanced for on-site exploration.

## Figures and Tables

**Table 1:** The composition and concentration of the gas mixture.

Components	Concentrations (ppm)								
samples	1	2	3	4	5	6	7	8	9
N_2_	balance gas	balance gas	balance gas	balance gas	5150	10,200	balance gas	balance gas	balance gas
H_2_	994.9	2086	9999	19,635	2010	5100	492	5030	50,000
CH_4_	535.3	2076	49,992	99,662	1970	4900	102	998	9950
CO_2_	1027.8	1928	51,050	99,662	1970	4820	499	1510	10,000
O_2_	0	0	0	0	0	0	0	0	0
Ar	0	0	0	0	balance gas	balance gas	0	0	0
Components	Concentrations (ppm)								
samples	10	11	12	13	14	15	16	17	18
N_2_	balance gas	50,100	10^5^	301,000	0	0	0	0	0
H_2_	0	0	0	0	10^6^	0	0	0	0
CH_4_	0	0	0	0	0	10^6^	0	0	0
CO_2_	0	balance gas	balance gas	balance gas	0	0	10^6^	0	0
O_2_	149,708	2000	10,000	50,100	0	0	0	10^6^	0
Ar	0	0	0	0	0	0	0	0	10^6^

**Table 2:** LOD values of various gas components.

Sample	Characteristic Peak (cm^−1^)	Scattering Cross-Section	LOD (ppm)
CH_4_	2917	6.0	3.14
CO_2_	1387	1.1	34.79
H_2_	4156	2.0	17.90
N_2_	2331	1.0	26.96
O_2_	1555	1.0	35.17

## Data Availability

Data are contained within the article.
